# Presynaptic pH and vesicle fusion in *Drosophila* larvae neurones

**DOI:** 10.1002/syn.21678

**Published:** 2014-05-07

**Authors:** Lesley Caldwell, Peter Harries, Sebastian Sydlik, Christof J Schwiening

**Affiliations:** Department of Physiology Development and Neuroscience, University of CambridgeDowning Street, Cambridge, CB2 3EG, United Kingdom

**Keywords:** exocytosis, neuromuscular junction, intracellular pH, Na^+^/H^+^ exchanger, synaptic transmission

## Abstract

Both intracellular pH (pH_i_) and synaptic cleft pH change during neuronal activity yet little is known about how these pH shifts might affect synaptic transmission by influencing vesicle fusion. To address this we imaged pH- and Ca^2+^-sensitive fluorescent indicators (HPTS, Oregon green) in boutons at neuromuscular junctions. Electrical stimulation of motor nerves evoked presynaptic Ca^2+^_i_ rises and pH_i_ falls (∼0.1 pH units) followed by recovery of both Ca^2+^_i_ and pH_i_. The plasma-membrane calcium ATPase (PMCA) inhibitor, 5(6)-carboxyeosin diacetate, slowed both the calcium recovery and the acidification. To investigate a possible calcium-independent role for the pH_i_ shifts in modulating vesicle fusion we recorded post-synaptic miniature end-plate potential (mEPP) and current (mEPC) frequency in Ca^2+^-free solution. Acidification by propionate superfusion, NH_4_^+^ withdrawal, or the inhibition of acid extrusion on the Na^+^/H^+^ exchanger (NHE) induced a rise in miniature frequency. Furthermore, the inhibition of acid extrusion enhanced the rise induced by propionate addition and NH_4_^+^ removal. In the presence of NH_4_^+^, 10 out of 23 cells showed, after a delay, one or more rises in miniature frequency. These findings suggest that Ca^2+^-dependent pH_i_ shifts, caused by the PMCA and regulated by NHE, may stimulate vesicle release. Furthermore, in the presence of membrane permeant buffers, exocytosed acid or its equivalents may enhance release through positive feedback. This hitherto neglected pH signalling, and the potential feedback role of vesicular acid, could explain some important neuronal excitability changes associated with altered pH and its buffering. **Synapse 67:729–740, 2013**.

## INTRODUCTION

Ca^2+^ has a pivotal role in vesicle release (Douglas, 1986; Katz and Miledi, 1967). However, there is not yet a complete understanding of the molecular mechanisms that couple Ca^2+^ influx to neurotransmitter release (Augustine, 2001; Chapman, 2008; van den Boggaart and Jahn, 2011), and there is some evidence for an involvement of calcium-independent mechanisms (Drapeau and Nachshen, 1988; Parnas et al., 2000). Many studies have focussed on the potential Ca^2+^ binding proteins associated with vesicles (Daily et al., 2010; Hay, 2007; Johnson and Chapman, 2010; Tang et al., 2006) but little attention has been paid to the possible role of presynaptic calcium-dependent intracellular acidifications (Wemmie et al., 2008; Zhang et al., 2010). The plasma-membrane calcium ATPase (PMCA) is known to produce transient acid shifts in neurones (Schwiening et al., 1993; Schwiening and Willoughby, 2002; Trapp et al., 1996) through the counter-transport of protons into cells. The intimate relationship between voltage-gated Ca^2+^ channels, the PMCA, and vesicle release sites (Juhaszova et al., 2000) and the multitude of potential targets for intracellular pH (pH_i_) shifts leads us to consider the possibility that the PMCA may have a role in shaping vesicle release. Previous work (Chen et al., 1998; Drapeau and Nachshen, 1988; Ohki and Arnold, 1990; Rocha et al., 2008; Trudeau et al., 1999) has demonstrated that vesicle fusion can be modulated by pH_i_, but there is no evidence of endogenous mechanisms that could provide pH_i_ changes that might influence fusion probability.

There have been several studies showing effects of extracellular pH transients (Sandstrom, 2011), cleft pH transients (Dietrich and Morad, 2010), or changes in vesicle pH (Camacho et al., 1996) on the speed and number of vesicles released, but the mechanism by which they act remains unclear. Both indirect studies of pH_i_ in synaptosomes (Jang et al., 2006; Sauvaigo et al., 1984) and direct measurements (Jean et al., 1985; Nashchen and Drapeau, 1988; Sánchez-Armass et al., 1994) have identified the presence of the acid extruder Na^+^/H^+^ exchanger (NHE) in synaptosomes. There have been two studies of presynaptic pH_i_ transients (Rossano et al., 2013; Zhang et al., 2010). Both studies show that stimulation can result in an initial acidification, although prolonged stimulation of mouse terminals showed a considerable regional heterogeneity of responses (see Fig. S3 in Zhang et al., 2010).

*Drosophila melanogaster* larvae motoneurone terminals have a high surface area to volume ratio and have large, fast Ca^2+^_i_ transients during stimulation (Macleod et al., 2002). Since Ca^2+^ extrusion is primarily on the PMCA (Lnenicka et al., 2006), pH_i_ shifts occur (Rossano et al., 2013). Here, we have investigated Ca^2+^ and pH_i_ transients, at the neuromuscular junction (NMJ), using pH and calcium-sensitive fluorescent indicators imaged on a confocal microscope during electrically evoked nerve stimulation. We then sought to investigate the direct (calcium-independent) effects of pH_i_ on vesicle release by removing extracellular calcium. Although evoked release is blocked by the absence of calcium, we were able to record changes in spontaneous mEPP frequency during a range of maneuvers that alter pH_i_.

## MATERIALS AND METHODS

### Presynaptic pH_i_ and Ca^2+^_i_ measurements

*Drosophila* larvae (wild-type Canton S) were raised on corn-meal agar with dry yeast at room temperature. Larvae (wandering third instar) were dissected (Jan and Jan, 1976) in Schneider's insect medium (Sigma) and pinned onto the Sylgard (Dow Corning) base of a 0.5 ml open chamber. The dissection and loading of fluorescent dyes was performed as described by Rossano and Macleod (2007). Briefly, efferent hemi-segment motor nerves were cut individually and several were drawn into a snug-fitting suction pipette. Suction pipettes were pulled from thin-walled borosilicate glass (GC100-T; Harvard Apparatus, UK) with tips cut to ∼300 μm with a ceramic tile (Composite Metal Services, UK), then polished to ∼12 μm internal diameter using a home-made gas microforge (Schwiening and Caldwell, 2008). Indicators (10,000 MW dextran-conjugated HPTS and Oregon Green BAPTA-1 (OGB-1); Molecular Probes, USA, ∼1.25 mM final concentration) were microperfused onto the nerve within 5 min of cutting the nerve using thin plastic tubing (∼200 μm diameter) inserted into the back of the pipette. Dye loading was carried out in Schneider's medium (5.4 mM Ca^2+^) and calcium-chelating dyes were removed after 40 min. Following the dye loading, nerves were left for 3–4 h, with solution changes every 45 mins, before recording (Rossano and Macleod, 2007). Immediately prior to imaging, Schneider's medium was replaced with the Hemolymph-Like No.6 (HL6; 0 mM HCO_3_^−^_,_ 15 mM N,N-Bis(2-hydroxyethyl)-2-aminoethanesulfonic acid (BES), pH adjusted to 7.20 with NaOH (Macleod at al., 2002). The HL6 also contained 7 mM glutamate, which desensitizes postsynaptic glutamate receptors (GluRs), and low 0.5 mM Ca^2+^, which inhibited muscle contraction.

Stimulation-evoked pH_i_ and [Ca^2+^]_i_ changes were recorded (Zeiss LSM510, 40× water-immersion objective, Germany) from *Drosophila* motornerve terminals as changes in fluorescence (500–600 nm) during excitation with the 488 nm line of an argon laser (50% power; PMT ∼800 V which results in bright and highly pH-sensitive fluorescence). Laser power (intensity and time) was minimized to avoid indicator photo bleaching. 2.5 V, 0.3 ms, 80 Hz, 2 s long trains were applied to the nerve by an isolated stimulator (DS2A; Digitimer Ltd., UK) through the suction pipette. Pixel-based analysis of fluorescence was performed using a specially written Visual Basic program to extract data from manually drawn regions of interest (ROI). Background-subtracted fluorescence intensities were normalised against *F*_o_, the mean intensity at steady-state prior to stimulation. Since images were collected at the maximum possible frame-rate, digital Gaussian low-pass filters (*τ* 0.06–0.3 s) were used, as appropriate, to reduce high-frequency noise. Movement in the *x–y* plane was corrected for by shifting key images within the time series and linear interpolation of drift between the key images. Relative HPTS fluorescence shifts were calibrated for ΔpH by assuming a resting pH_i_ of ∼7.18 (see Results section) for Is and Ib type boutons (Johansen et al., 1989) and pK_HPTS_ of 7.18, using a simple form of the Grynkiewicz equation modified for use with HPTS when excited at 488 nm (Schwiening and Willoughby's (2002) Equation 1). The coplanar Ib terminal on muscle 4 was chosen when viable to allow regional fluorescence signals from 2 to 3 distal boutons to be visualised and averaged together in a single ROI to reduce noise. The acceptance criteria for analysis were as follows: detectable dye loading, the absence of spontaneous or evoked muscle contractions, and the presence of stimulation-induced pH_i_ or Ca^2+^_i_ transients prior to addition of any pharmacological drugs.

Pharmacological agents included sodium propionate, procaine hydrochloride, NH_4_Cl, trimethylamine hydrochloride (TMA), amiloride, ethyl-isopropyl amiloride (EIPA), 4-acetamido, 4′-isothiocyanato-2,2′-stilbene disulfonate (SITS) (Sigma, UK), 5(6)-carboxyeosin (BioChemika), and 5% CO_2_/95% O_2_ (BOC, UK). The pH of the solutions was measured and reset after compounds were added and they were covered, when necessary, to minimize the loss of volatile components. During the recording of mEPPs and miniature end plate currents (mEPCs), the baths were continually superfused at a rate 1–2 ml min^−1^ using a gravity-fed superfusion system. During the imaging of activity-evoked Ca^2+^ and pH_i_ shifts, the superfusion was temporarily stopped to improve image stability.

### Recording of miniature end plate events

mEPPs (Fatt and Katz, 1952) and mEPCs were generally recorded from muscle 4 or 6 using 3M KCl-filled microelectrodes (borosilicate glass, GC-100F; Clark Electromedical Instruments; 30–40 MΩ resistance). Two-electrode voltage clamp was used to record mEPCs. Recordings of membrane potential (*E*_m_) and current during voltage clamping were performed using an Axoclamp 2B (HS-2A headstages; Axon Instruments, USA) filtered using a 300 Hz low-pass analogue filter and amplified 1,000 times before digitization at 1 kHz using a CED 1401 and Spike2 (Cambridge Electronic Design, UK). HL3 Ringer was used for dissections and recordings of miniatures and contained (in mM) 70 NaCl, 20 MgCl_2_, 5 KCl, 15 BES, 115 sucrose, and 5 Trehalose (pH 7.20) with no added calcium (Stewart et al., 1994). mEPCs and mEPPs were detected by their rapid initial transient using a threshold criterium on a low-pass differential filtered trace of either clamp current or *E*_m_, respectively (Spike 2). Figure 3a shows an example for the detection of mEPPs. Mean frequency was calculated in 2.5 s time bins, with a subsequent 4-point running average. Whenever mEPC or mEPP frequency rose or fell, the clamp current or *E*_m_ were inspected to rule out miscounts. Experiments were discarded if the addition of compounds resulted in depolarizations that prevented the accurate counting of mEPPs and, in addition, the experiments were repeated using voltage clamp. Other indicators of miscounting were used; high-frequency baseline (>5 Hz), unprovoked sudden changes in mEPCs or mEPP amplitude, frequency and changes in the ratio between the mEPCs/mEPP SD and mean. The adherence to the Poisson distribution (Kriebel et al., 1976) was used to identify electrical and mechanical noise as they drove the ratio of SD to mean below 1.

### Data analysis

Straight lines and exponentials (scalar × e^−time/^*^τ^* + constant, where *τ* is the time constant and –scalar/τ is the initial rate) were fitted by least squares (Spike 2 and using the Solver in Excel).

### Statistics

Statistical significance (*P* < 0.05) was determined using Student's t-test (two-sample, two-tailed, unequal variance data). * is *P* < 0.05 and ** is *P* < 0.01. Results are presented as mean ± standard error, unless otherwise specified; *n* represents the number of separate larvae, not different cells from the same preparation.

## RESULTS

### pH_i_ and Ca^2+^_i_ measurements at the NMJ

**Fig 1:**
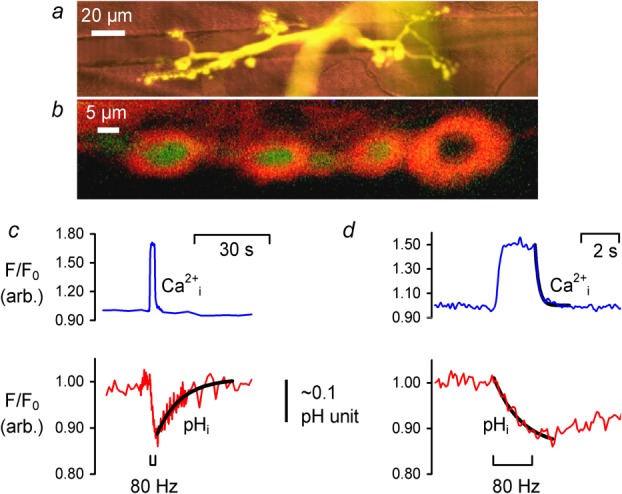
Fluorescence imaging of *Drosophila* larva neuromuscular junctions in 0.5 mM Ca^2+^. **a** 488 nm excited fluorescence image of motoneurone terminals forward-filled with HPTS (shown in yellow, average of 214 frames) superimposed on the transmitted reference image of body-wall muscle fibres 7 and 6 (red, average of 74 frames). **b** Motoneurone boutons forward-filled with HPTS (shown in green) and extracellular plasmalemmal surfaces stained with Sulforhodamine B (SRB, 565 nm excitation, >586 nm emission shown in red). The SRB staining surrounding boutons was 1.39±0.24 μm thick (n=2, 4 NMJs, pinhole 1.2 airy units) corresponding to the infoldings of the postsynaptic target muscle membrane (Lnenicka et al., 2006). **c** Aligned F/F_0_ OGB-1 (Ca^2+^_i_, blue trace) and HPTS (pH_i_, red trace) fluorescence intensities during 80Hz 2 s long stimulus trains from ROIs placed over distal nerve bouton regions. The pH transient is overlaid with an exponential fit from which pH_i_ τ_recovery_ was extracted. **d** Aligned Ca^2+^_i_ (blue) and pH_i_ (red) transients at higher temporal resolution. The plots show exponential fits to the Ca^2+^ recovery and acidification, from which [Ca^2+^]_i_ τ_recovery_ and pH_i_ τ_acidification_ were extracted.

Dextran bound fluorescent indicators (HPTS and OGB-1) labelled both nerves and associated junctions (Fig. 1a), although not all boutons (as indicated by surrounding Sulforhodamine B fluorescence) contained visible 488 nm (acid quenched) HPTS fluorescence (Fig. 1b). Ratiometric imaging (Leica SP5 confocal 488/405 nm HPTS fluorescence, data not shown) showed no significant difference between resting pH_i_ (7.18 ± 0.16, mean ± SD; *n* = 4 larvae, 5 segmental nerves, 13 NMJ) in the two largest NMJ types designated as type-Ib (big) and type-Is (small) boutons (Johansen et al., 1989).

Top traces in Figures 1c and 1d show typical recordings of Ca^2+^-sensitive fluorescence (OGB-1 dextran), during 80 Hz, 2 s stimulus. Ca^2+^_i_ increased rapidly upon stimulation before reaching a plateau (50–80% increase in fluorescence) consistent with stimulation-evoked Ca^2+^ influx through voltage-gated Ca^2+^ channels. Following stimulation, Ca^2+^_i_ recovered rapidly. The stimulation-evoked Ca^2+^_i_ transients were maximal at stimulation frequencies ≥60 Hz and were present in ∼90% of the NMJs imaged (*n* ≈ 50). Ca^2+^_i_ shifts were heterogeneous, with distal boutons typically displaying greater Ca^2+^_i_ rises with faster kinetics relative to proximal axonal regions. The mean shifts showed a 0.64 ± 0.02 Δ*F*/*F*_o_ increase in OGB-1 fluorescence (*n* = 6, *P* < 0.001) with a recovery time constant, *τ*_recovery_, of 0.15 ± 0.02 s. The bottom traces in Figures 1c and 1d show the stimulation-evoked pH-sensitive fluorescence decreases (HPTS) of ∼11% consistent with transient intracellular acidifications. Following stimulation, HPTS fluorescence recovered, however, the extent of the recovery varied between junctions. On average, stimulation induced a 0.09 ± 0.03 Δ*F*/*F*_o_ decrease in HPTS fluorescence (*n* = 6, *P* < 0.05). Using a single wavelength calibration, assuming a resting pH_i_ of ∼7.18 in type-Is and Ib boutons, this Δ*F*/*F*_o_ decrease equates to an intracellular acidification of ∼0.1 ΔpH units. The mean acidification time constant, *τ*_acidification_, was 1.25 ± 0.13 s (*n* = 6). pH_i_ transients recovered with mean time constant, *τ*_recovery_, ∼9 times greater (11.0 ± 2.0 s).

Although the latency of the pH_i_ and Ca^2+^_i_ transients appeared similar, the rate of rise of calcium was greater than the acidification rate (Fig. 1d), and it began to recover as soon as the stimulus stopped. The recovery from the acidification, however, only began as Ca^2+^_i_ reached baseline levels. Ca^2+^_i_ recovery was also faster than that of pH_i_. These observations are consistent with Ca^2+^ entry through Ca^2+^ channels and extrusion by the PMCA in exchange for H^+^, leading to acidification of the nerve terminal.

**Fig 2:**
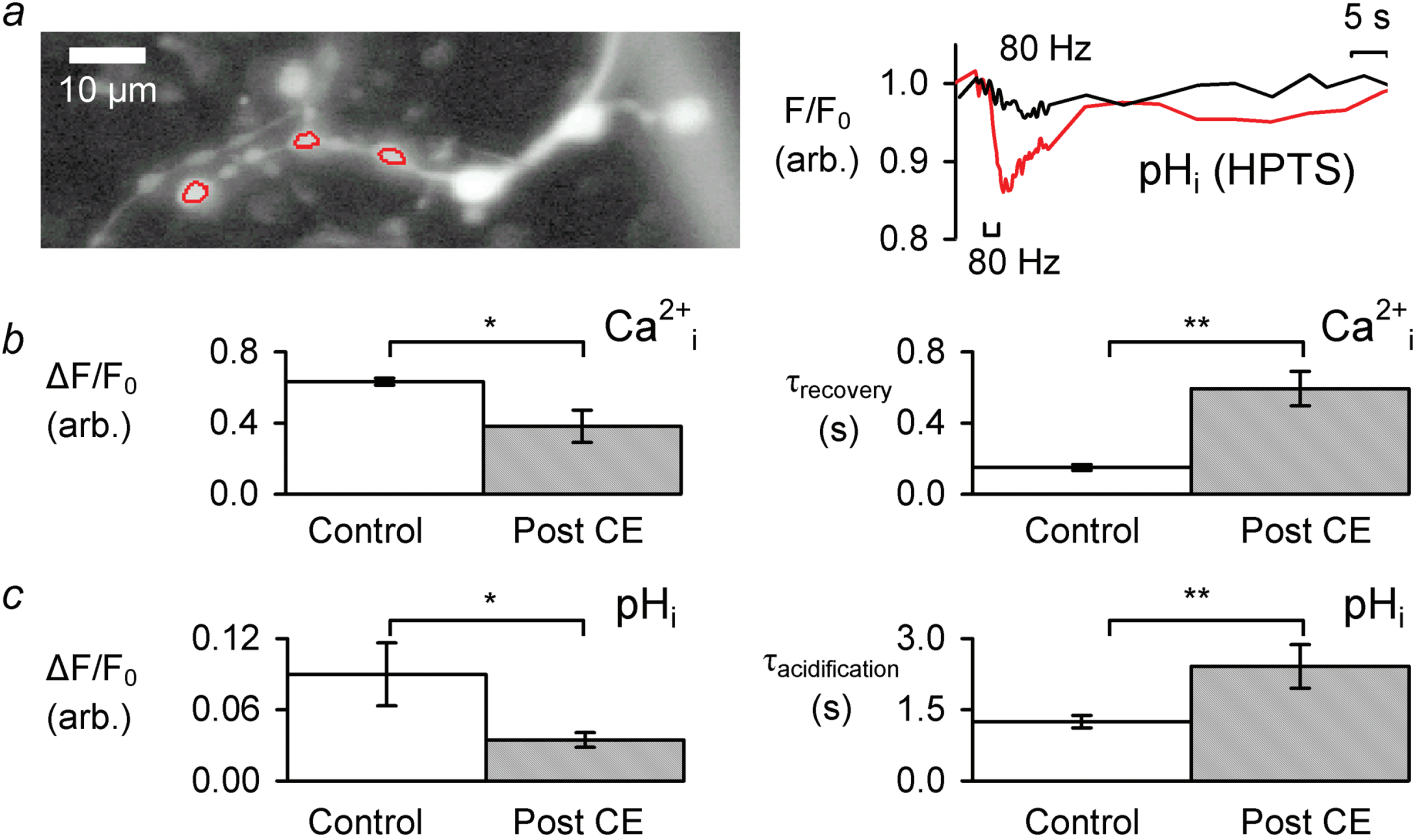
Effect of 5 μM carboxyeosin on Ca^2+^ and pH_i_ transients. **a** 488 nm excited HPTS fluorescence reference image of NMJ boutons overlaid with an ROI covering 3 boutons (average of 27 frames) and superimposed plots of F/F_0_ fluorescence intensity during stimulus trains. The red trace is in control and the black trace is after 5 μM CE has been removed. Intervals between frames with no laser illumination were altered to allow fast changes to be captured whilst minimizing dye photodamage. **b** Ca^2+^_i_ transient ΔF/F_o_ and τ_recovery_ prior to CE application and 20 min after 5 μM CE was removed. **c** pH_i_ transient ΔF/F_o_ and τ_acidification_ prior to CE application and after CE removal.

To test this hypothesis the contribution of the PMCA to these transients was assessed using the inhibitor 5(6)-carboxyeosin diacetate (CE) (Gatto and Milanick, 1993; Balasubramanyam and Gardner, 1995) which, at low concentrations (*K*_d_ ∼ 50 nM, which is approximately 10,000 times lower than the HPTS concentration), does not interfere with HPTS fluorescence (Schwiening, 1997). A total of 40 min application of 5 μM CE (followed by wash-off) reduced the stimulation-evoked transient increase in Δ*F*/*F*_o_ OGB-1 fluorescence by 39 ± 13% (*n* = 5, *P* < 0.05) and increased the [Ca^2+^]_i_ *τ*_recovery_ by 339 ± 116% (Fig. 2b, *n* = 5, *P* < 0.01). Higher concentrations of CE (10–20 µM, 15 min, *n* = 3) abolished Ca^2+^_i_ transients. Figure 2a shows an HPTS-loaded nerve and, in red, boutons from which fluorescence was plotted. The pH-sensitive fluorescence transient was ∼0.13 Δ*F*/*F*_o_ before, and ∼0.04 Δ*F*/*F*_o_ after exposure to CE and took longer period to reach its peak. On average, CE reduced the amplitude of the Δ*F*/*F*_o_ decrease in HPTS fluorescence by 59 ± 7% (*n* = 5, *P* < 0.05 paired t-test, Fig. 2c). pH_i_ *τ*_acidification_ increased by 137 ± 47% after CE application (*n* = 5, *P* < 0.01), consistent with a reduction in pH_i_ transient acidification rate. The slowing of both the Ca^2+^ recovery and the pH acidification, as well as the reduction in the size of the pH_i_ transient by CE are again consistent with the PMCA acting to extrude Ca^2+^ in exchange for H^+^.

**Fig 3:**
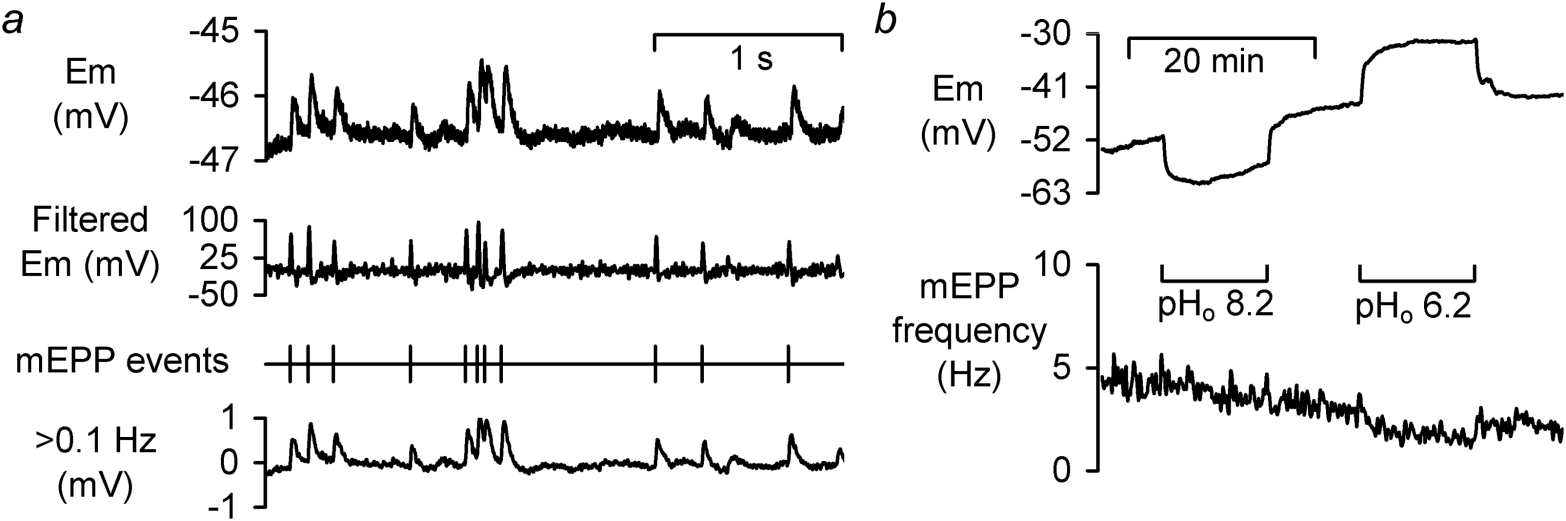
Postsynaptic membrane potential and mEPPs. **a** Traces of E_m_, low pass differential of E_m_ (used for counting), the derived mEPP markers, and the AC component of E_m_ trace used to aid visualization of mEPP count accuracy. **b** E_m_ and mEPP frequency during exposure to alkaline and acidic pH_o_.

### Effect of pH on mEPP frequency

To investigate any direct, Ca^2+^-independent, role for the pH_i_ transients, and to elucidate the pH_i_ regulating mechanisms, we recorded muscle membrane potential (*E*_m_) and calculated spontaneous mEPP (Fatt and Katz, 1952) and mEPC frequency in the absence of both electrical stimulation and Ca^2+^. Figure 3a shows typical raw and filtered *E*_m_ data and the detected mEPPs. Resting excitatory postsynaptic mEPP frequency varied between 1.4 and 5.6 Hz (mean 3.13 ± 0.18 Hz, *n* = 137). Unlike evoked-end plate potentials in the presence of HCO_3_^-^ (Sandstrom, 2011) alteration of pH_o_ (by ∼1 pH unit), in the absence of weak acids or bases, caused small changes in absolute mEPP frequency (Fig. 3b) with ∼10 mV changes in muscle membrane potential. Extracellular acidification to pH 6.2, caused a 0.84 ± 0.1 Hz (32 ± 7%) decline in mEPP frequency (*n* = 4, *P* < 0.01). Extracellular alkalinisation to pH 8.2 had no significant effect (0.22 ± 0.13 Hz rise in mEPP frequency, *n* = 4, *P* > 0.09).

**Fig 4:**
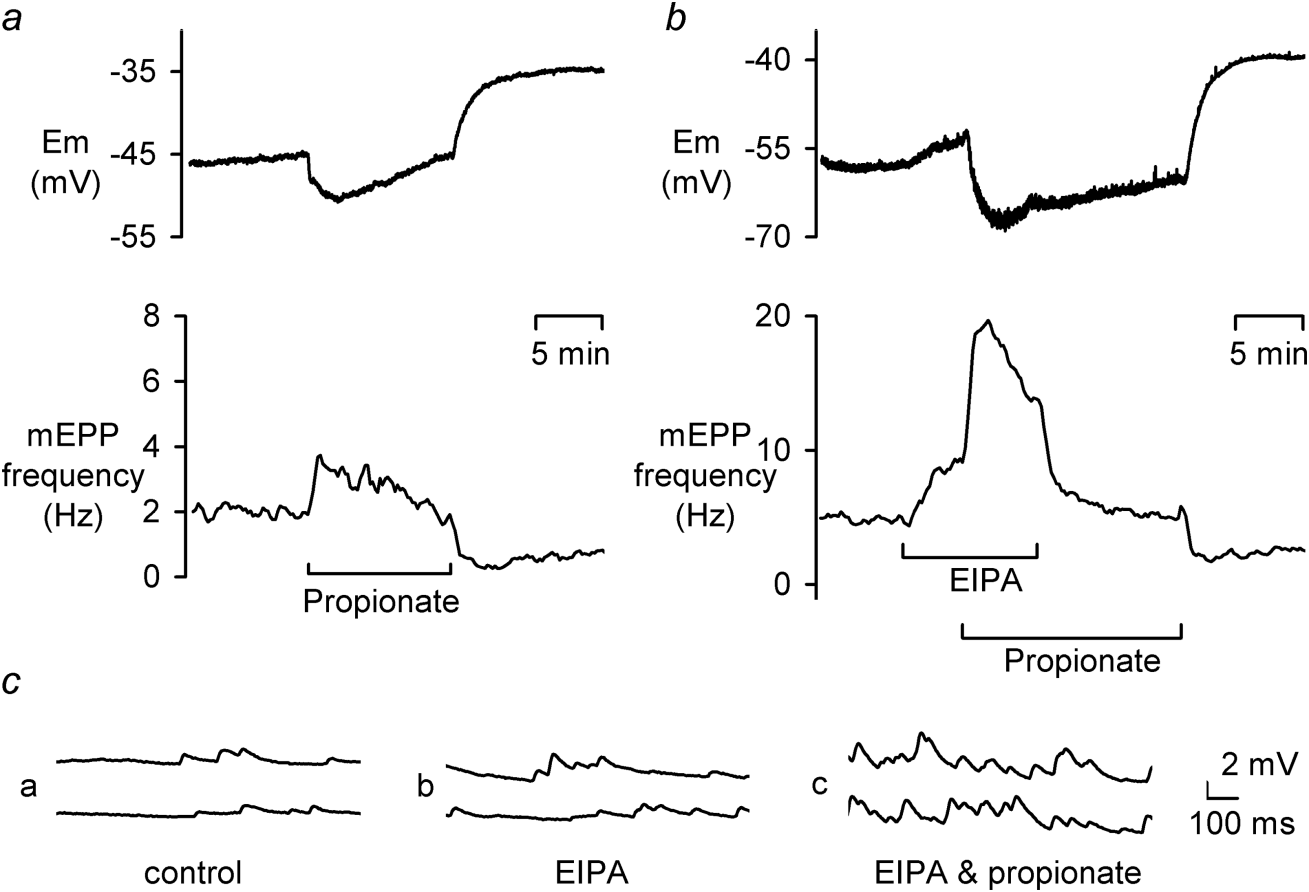
Effect of 50 μM EIPA on 20 mM propionate-induced mEPP frequency transients. **a** E_m_ and mEPP frequency during 10 min application of 20 mM propionate. **b** E_m_ and mEPP frequency during 50 μM EIPA and 20 mM propionate application. **c** mEPPs at high time resolution from (**b**).

Although relatively large changes in pH_o_ were only able to depress mEPP frequency, smaller intracellular acidifications were effective at raising mEPP frequency; Figure 4a shows a typical trace during superfusion with 20 mM propionate. mEPP frequency rose from ∼1.9 Hz to ∼3.5 Hz (mean 2.41 ± 0.52 Hz to 4.80 ± 1.36 Hz, an 87 ± 18% increase, *n* = 7, *P* < 0.05), followed by an immediate but slower decline in mEPP frequency. Upon propionate removal, mEPP frequency always dropped below initial levels. The *E*_m_ changes on propionate addition and removal follow with the predicted waveforms of pH_i_ on acidification of muscle (Bountra and Vaughan-Jones, 1989). These changes in mEPP frequency also appear to be driven by pH_i_ since they follow the same waveform as the classical pH_i_ changes caused by the addition and removal of a weak acid. Application of propionate is known to causes an initial acidification which gradually recovers (pH_i_ regulation). Subsequent removal of propionate then causes a rebound alkalinisation beyond the initial resting pH. The mEPP frequency changes follow this waveform and, as expected from a high surface area to volume ratio region, are faster than those of the muscle membrane potential. Since pH_i_, in areas of high surface area to volume ratio, is known to be very sensitive to changes in transmembrane fluxes of proton (Schwiening and Willoughby, 2002), pH-sensitive mEPP frequency should also be highly sensitive to inhibition of pH_i_ regulation.

In an attempt to test this, we used the NHE inhibitor EIPA (5–100 μM). EIPA concentrations ≥20 μM produced a reversible, dose-dependent increase in mEPP frequency with no significant change in their amplitude. As shown in Figure 4, 50 μM EIPA caused mEPP frequency to double (mean data 3.05 ± 0.76 to 5.93 ± 1.58 Hz, a 94 ± 12% increase, *n* = 4, *P* < 0.05). Subsequent application of 20 mM propionate caused a further mEPP frequency rise to ∼19.5 Hz (on average 16.0 ± 3.1 Hz, *n* = 4). In the presence of EIPA, this propionate-induced mEPP frequency rise was significantly greater at 485 ± 107% (*n* = 4, *P* < 0.05) when compared to the 87 ± 18% (*n* = 7) rise in EIPA-free solution (*P* < 0.001). To quantify the effect of EIPA, we have calculated the percentage change in mEPP frequency on EIPA removal. This allows a direct comparison of a potential pH_i_-sensitive process at identical pH_i_. To do this, exponentials were fitted to the mEPP frequency recovery data in EIPA and following EIPA removal from which the final rate of recovery in EIPA and the initial rate of recovery following EIPA removal (both at the same mEPP frequency) were calculated. This revealed that EIPA slowed (in the presence of propionate) the decline in mEPP frequency by ∼89% (−17.6 ± 6.5 mHz s^−1^ in EIPA to −167.2 ± 34.5 mHz s^−1^ on EIPA removal, *n* = 4, *P* < 0.05). It is possible that changes in muscle *E*_m_ may have influenced the ability to detect mEPPs; however, it is unlikely to explain the frequency changes seen here. First, plots of mEPP frequency against *E*_m_ during weak acid addition and removal show marked hysteresis due to the rapid kinetics of the mEPP frequency changes compared to *E*_m_ such that large mEPP frequency changes occurring with little change in *E*_m_. Furthermore, we repeated the experiments under voltage clamp, recording mEPCs, and found similar results. For instance, on EIPA addition, mEPC frequency rose from 1.9 ± 0.6 Hz to 4.4 ± 0.7 Hz (*n* = 6), ∼0.5 Hz lower and not significantly different from the mEPP frequency values. Similarly, propionate addition, in EIPA, caused mEPC frequency to increase to 14.4 ± 0.5 Hz (*n* = 6), only slightly lower than the 16.0 ± 3.1 Hz value when recording mEPPs. Thus, there is little evidence to support the notion that changes in muscle *E*_m_ underlie the reported changes in mEPP frequency.

Although these pH_i_-induced changes in miniature frequency were recorded in solutions containing no added Ca^2+^, it is possible that intracellular stores may have contained sufficient Ca^2+^ such that acidification might cause Ca^2+^ release thereby stimulating fusion. To test this, we compared mEPC frequency in preparations that were first depleted of Ca^2+^ by exposure to 0 Ca^2+^/1 mM EGTA during which 10 mM caffeine was repeatedly applied with preparations that were bathed in 5 mM Ca^2+^. The mEPC frequency rise induced by propionate, in the presence of EIPA, was not significantly different between these two conditions (12.4 ± 1.3 Hz, *n* = 8 in the Ca^2+^-depleted state compared to 9.7 ± 1.8 Hz *n* = 4 in 5 mM Ca^2+^, *P* > 0.2).

To test for the presence of anion-dependent pH_i_ regulating mechanisms, SITS was applied. This was done in the absence of added HCO_3_^−^ since SITS-sensitive anion-dependent pH_i_ regulating mechanisms appear to operate in insects in the absence of added HCO_3_^−^ (Romero et al., 2000; Schwiening and Thomas, 1992). SITS caused no significant change in mEPP frequency (control 2.14 ± 0.20 Hz to 1.40 ± 0.32 Hz in 200 μM SITS, *n* = 4, *P* > 0.1). To test the contribution of anion-dependent pH_i_ regulating mechanisms following acidification, propionate was applied in the presence of both 50 μM SITS and 50 μM EIPA. In the presence of EIPA, SITS again had no significant effect on mEPP frequency (*n* = 6), it also did not affect the size of the frequency rise induced by propionate (*n* = 6) or the rate at which mEPP frequency recovered following the propionate-induced frequency transient (*n* = 5). These results are consistent with NHE rather than a HCO_3_^−^-dependent mechanism being the main pH regulating mechanism active both at rest and during recovery from an acid load. However, in the presence of SITS, the propionate-induced frequency rise was followed by a plateau phase, or an additional slower rise, prior to recovery (data not shown). A linear fit was made to 2 min of the mEPP frequency data from ∼100 s after propionate addition. This yielded a significant difference with an mEPP frequency decline of 10.3 ± 5.3 mHz s^−1^ (*n* = 4) in the absence of SITS, compared to an mEPP frequency rise of 9.9 ± 5.6 mHz s^−1^ in the presence of SITS (*n* = 6, *P* < 0.05). Thus, it would appear that an SITS-sensitive pH_i_ regulating mechanism may be present, but it is inactive at resting pH_i_ and furthermore its ability to recover pH_i_ may be limited to just the initial period of acidification.

Application of 24 mM HCO_3_^−^/5% CO_2_ (0 mM BES) had no obvious effect on mEPP frequency (*n* = 4). The same protocols shown for propionate and EIPA addition in Figure 4, as well as SITS applications, were repeated in the presence of 24 mM HCO_3_^−^/5% CO_2_. No significant differences were seen between percentage mEPP frequency rise caused by EIPA (*n* = 3) or EIPA combined with SITS (*n* = 4) in the presence and absence of HCO_3_^−^. Also, in the presence of HCO_3_^−^ propionate addition, either in the presence of EIPA (*n* = 3) or EIPA and SITS (*n* = 4) produced similar frequency changes and initial recovery rates as in the absence of HCO_3_^−^. These results support the conclusion of Rossano et al. (2013) suggesting that there is little role for a HCO_3_^−^ or anion-dependent pH_i_ regulating mechanism at the NMJ.

### NH_4_^+^ prepulse technique

**Fig 5:**
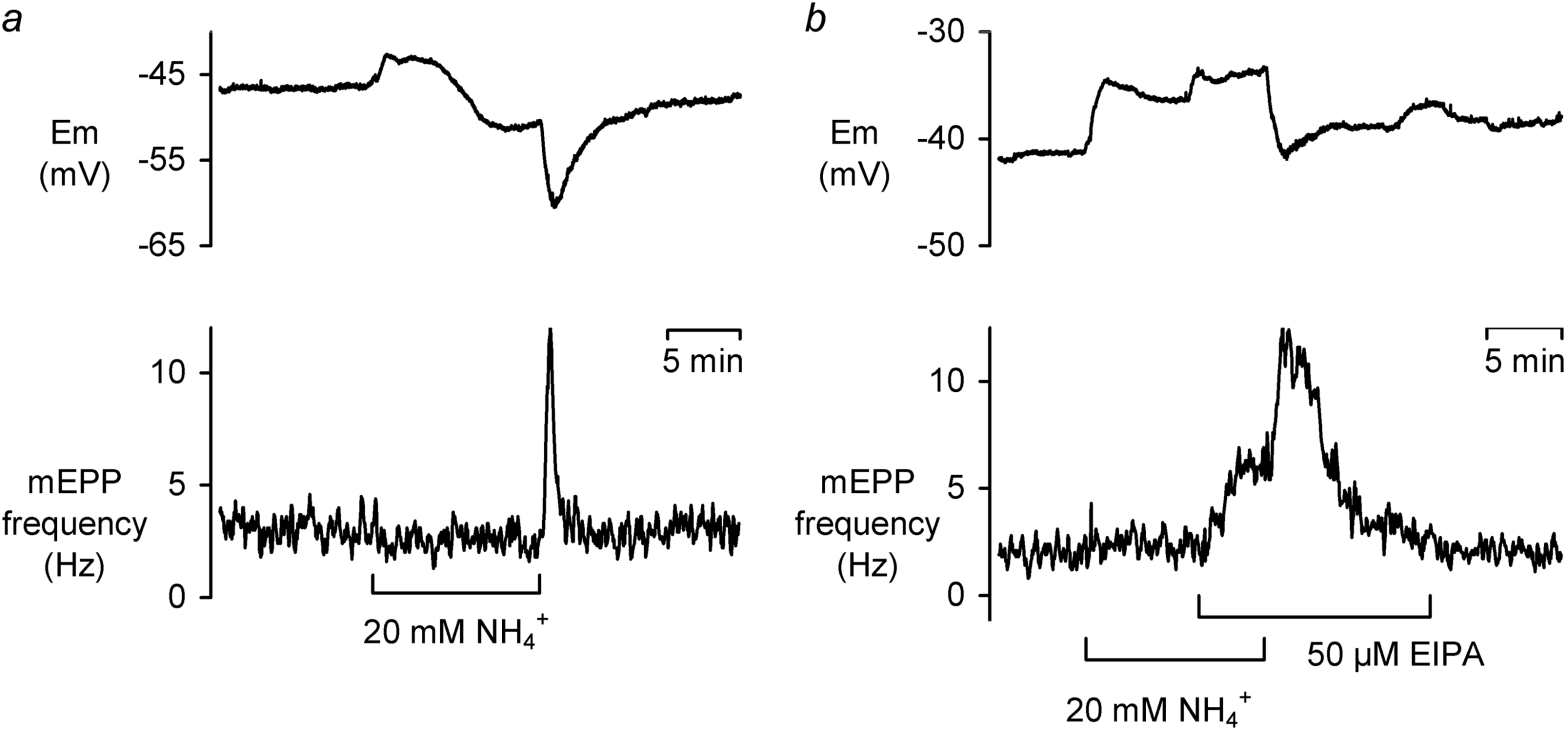
The effect of 50 μM EIPA on 20 mM NH_4_^+^ prepulse-induced mEPP frequency transients. **a** E_m_ and mEPP frequency upon NH_4_^+^ removal in control solution. **b** E_m_ and mEPP frequency upon NH_4_^+^ removal in the presence of EIPA.

To confirm that the propionate-induced effects on mEPP frequency were pH_i_ related, an alternative means of inducing presynaptic acidification was sought. The NH_4_^+^ prepulse technique (Boron and De Weer, 1976) consisting of application and subsequent removal of weak base NH_4_^+^, has the advantage of acidifying cells on return to the control solution. Figure 5 shows examples of NH_4_^+^ application for 10 min, followed by removal in the absence (Fig. 5a) and presence (Fig. 5b) of EIPA. No immediate change in mEPP frequency was observed upon NH_4_^+^ exposure; however, later frequency alterations were seen in some preparations (see following section). On NH_4_^+^ removal, a transient rise in mEPP frequency was observed.

20 mM NH_4_^+^ removal induced a transient mEPP frequency rise in four of nine preparations. Of the four showing a response, the average rise in mEPP frequency was from a baseline of 2.93 ± 0.31 Hz to 15.75 ± 2.66 Hz (*n* = 4, *P* < 0.05). The absence of mEPP frequency transients, on NH_4_^+^ removal, in some preparations led us to suspect that, in the absence of added buffer, pH_i_ regulation is so rapid and the relatively slow superfusion system may only be able to induce a small pH_i_ change. To test this, we slowed pH_i_ regulation with EIPA.

50 μM EIPA application, in the presence on NH_4_^+^, increased the mEPP frequency by 147 ± 15% (from 3.41 ± 0.60 to 8.45 ± 2.28 Hz, *n* = 6, *P* < 0.05). Subsequently, in the continued presence of 50 μM EIPA, NH_4_^+^ removal increased mEPP frequency to 18.72 ± 2.08 Hz in five of the six preparations. Once the nonresponding cell was removed, there was no significant difference between the relative frequency rise in the presence (559 ± 197%, *n* = 5, *P* < 0.01) or absence (459 ± 107%, *n* = 6, *P* < 0.05) of EIPA. There are two possible explanations for the failure to detect a higher peak mEPP frequency in the presence of EIPA. First, it is difficult to accurately determine mEPP frequency at very high release rates as the events begin to superimpose. To test this, we repeated the experiments under voltage clamp, recording mEPCs (which are faster events since they are not smoothed by the membrane capacitance), the frequency attained following NH_4_^+^ removal (17.0 ± 3.5 Hz, *n* = 5) was not significantly different. Second, it is also possible that the NH_4_^+^ removal induced rise in mEPP frequency is EIPA insensitive. To test this, we fitted exponentials to the recovery phase of the mEPP frequency data following the NH_4_^+^ removal, both in the presence and absence of EIPA. In the absence of EIPA, the initial rate of the frequency recovery was −434 ± 49 mHz s^−1^ (*n* = 4), and in the presence of EIPA, it was roughly a quarter of that rate: −107 ± 21 mHz s^−1^ (*n* = 4). This represents a ∼75% slowing (*P* < 0.05) of the decline in mEPP frequency induced by EIPA and suggests that mEPP frequency changes caused by either the addition of weak acid or the removal of weak base are regulated by NHE.

### Steady-state application of NH_4_^+^

**Fig 6:**
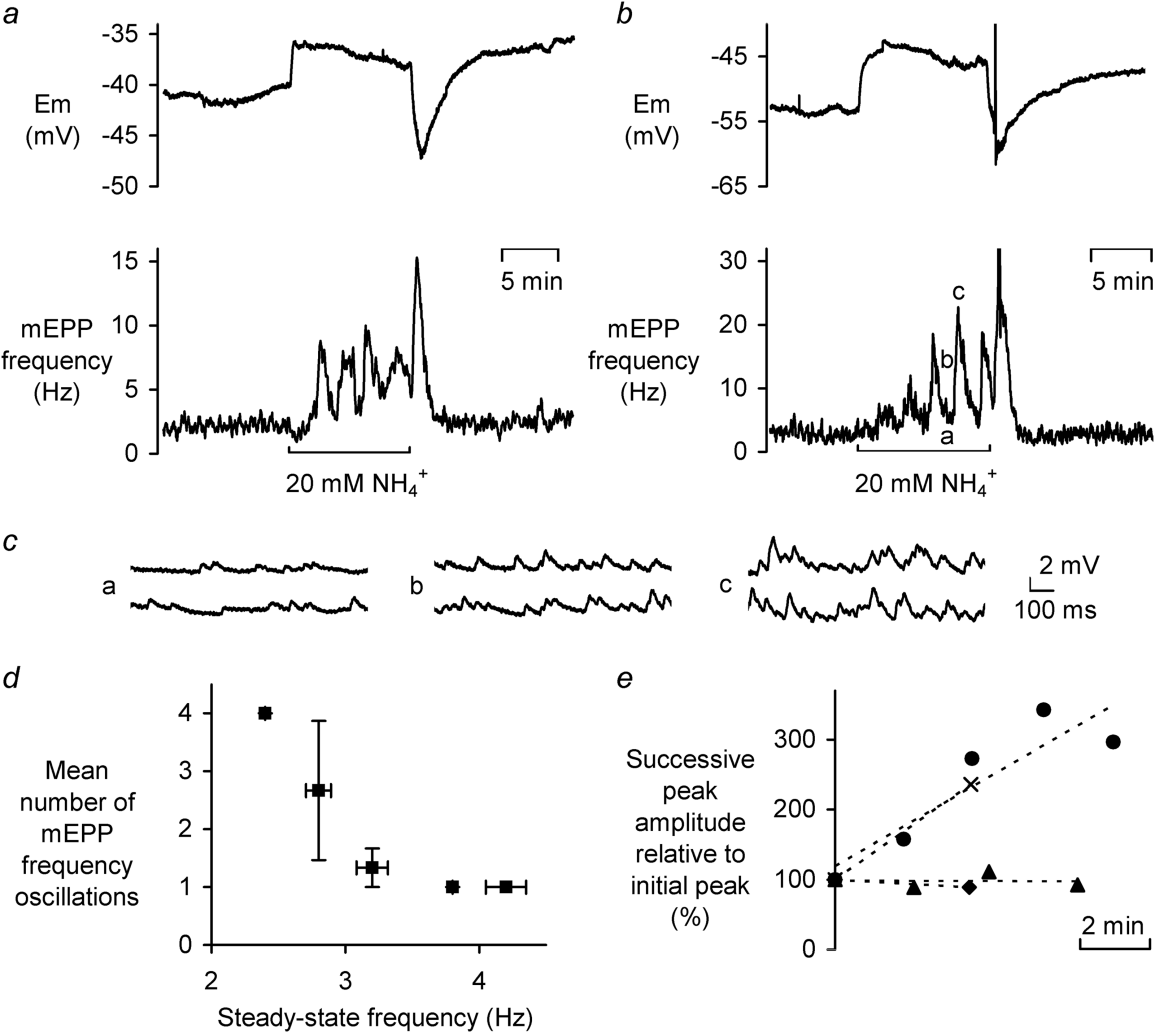
Effect of 20 mM NH_4_^+^ exposure on mEPP frequency. **a** E_m_ and mEPP frequency during the superfusion of NH_4_^+^ showing mEPP frequency oscillations. **b** E_m_ and mEPP frequency from another preparation during NH_4_^+^ exposure. The microelectrode became displaced from the muscle fibre, following 20 mM NH_4_^+^ removal, upon repositioning the recordings were unaffected. **c** mEPPs at high time resolution from **b** as indicated. **d** Steady-state baseline mEPP frequency prior to oscillations occurring during 10 min exposures to 20 mM NH_4_^+^ plotted against mean number of oscillations in resting mEPP frequency bins of 0.4 Hz width (n=1,3,3,1,2 respectively). Only preparations exhibiting oscillations were considered. **e** Successive oscillation amplitudes relative to that of the first NH_4_^+^-induced oscillation plotted against the time from NH_4_^+^ application (n=4, each preparation represented by a different symbol).

The stimulation of vesicle release by presynaptic acidification led us to consider the possibility that vesicular acid (Russell, 1984), released into the cleft, might have a positive feedback role if it could reenter the terminal. To test this, mEPPs were recorded in the continuous presence of the membrane permeant pH buffer NH_4_^+^, Figure 6 shows two such examples.

NH_4_^+^ exposure (*n* = 23) produced very little immediate change in mEPP baseline frequency; however, in 10 preparations, delayed large transient increases in mEPP frequency were observed. Some preparations only displayed one transient, while others, such as those shown in Figure 6, displayed several transients in an oscillatory manner. On average, mEPP frequency rose from 2.78 ± 0.25 Hz at steady state to 12.9 ± 1.6 Hz on the first oscillation (296 ± 43% increase). mEPP frequency transients of this amplitude were not seen in control solution in any experiment and all frequency transients were inspected to verify that the frequency increase was not due to a miscount of mEPPs (see Methods section).

Cells exhibiting multiple oscillations appeared to have relatively low resting mEPP frequencies. To analyse this further, we plotted the mean number of oscillations against the mean baseline mEPP frequency (Fig. 6d). Only cells with resting mEPP frequencies between 2.2 Hz and 4.3 Hz exhibited oscillations (or a single delayed mEPP frequency rise in the presence of NH_4_^+^), with those with the lowest resting mEPP frequency showing more oscillations.

The oscillations appeared to follow one of the two trends. Figure 6e shows the oscillation amplitudes, for four cells that exhibited multiple oscillations, as a percentage of the primary oscillation amplitude. The data from each preparation were fitted with a linear trendline. Two of the cells showed a second peak with increased amplitude relative to the initial peak, one of which showed more oscillations with progressively increasing amplitudes. Both of those cells showed a similar rate of increase in oscillation amplitude (∼30% min^−1^). The other two cells did not show increased amplitudes on subsequent oscillations, instead oscillation amplitudes remained stable over time.

The time between NH_4_^+^ addition to the start of the first oscillation varied from 14 to 352 s (mean 2.33 ± 0.59 min) and the time between oscillations was 151 ± 16 s. The oscillation time period for each experiment was relatively constant (e.g., 139 ± 7 s for the cell showing four transients and 119 ± 1 s for the cell showing five transients).

The effects of another base, TMA, were tested (data not shown). As with NH_4_^+^, exposure to 10 mM TMA for 6 min induced a reversible depolarization of the muscle membrane and caused no discernable change in mEPP frequency baseline; however, after a delay (45 ± 23 s), a transient frequency increase was observed in both preparations tested. The mean frequency rise was 1023 ± 34% (*n* = 2). These effects of TMA are consistent with the NH_4_^+^ experiments.

## DISCUSSION

### pH_i_ and Ca^2+^_i_ measurements at the NMJ

Our results confirm (Guerrero et al., 2005; Lnenicka et al., 2006; Macleod et al., 2002) that electrical stimulation induces transient increases in Ca^2+^-sensitive OGB-1 fluorescence in presynaptic regions of the NMJ. The resting pH_i_ (∼7.18) is similar to that previously reported in other neuronal preparations including *Drosophila* larvae nerve terminal using a genetically encoded pH indicator (Rossano et al., 2013) and locust neurones using ion-sensitive microelectrodes (Schwiening and Thomas, 1992). The evoked-acidifications (∼0.1 pH units) are similar to those reported by Rossano et al. (2013) at the same calcium concentration and to those reported in small postsynaptic regions in rat cerebellar Purkinje neurones (Willoughby and Schwiening, 2002) but appear to be larger than those reported by Zhang et al. (2010) using YFP in mouse motor nerve terminals. It is likely that our measurements underestimate the size of the near-membrane pH_i_ changes, not just as a result of the lower calcium concentrations, but since they are spatially and temportally filtered by the recording method. *In vivo*, during movement, it is likely that larger pH shifts occur in the terminal as a result of repetitive firing (Rossano et al., 2013).

The stimulation-induced pH_i_ transients started with no discernable delay, reached a peak around the time that Ca^2+^_i_ had recovered fully. Both the calcium recovery and the acidification were inhibited by CE supporting the involement of the PMCA, consistent with both the findings of Rossano et al. (2013) and the demonstration that *Drosophila* presynaptic terminals are rich in PMCA (Lnenicka et al., 2006). Indeed, the PMCA has been implicated in the extrusion of Ca^2+^ from other presynaptic regions (Morgans et al., 1998; Juhaszova et a., 2000).

The recovery of pH_i_ in *Drosophila* boutons has not previously been studied. The direct measurements of pH_i_ presented here show a high initial recovery rate (∼0.4 ΔpH_i_ min^−1^) almost 10 times faster than in locust neurone cell bodies (Schwiening and Thomas, 1992), but similar to that seen in rat Purkinje dendrites (Willoughby and Schwiening, 2002) and is consistent with the high surface area to volume ratio of the boutons.

### Modulation of mEPP frequency

Changes in pH_o_, larger than the steady-state changes expected physiologically, had little effect on mEPP frequency thus, it seems likely that vesicle release is not directly sensitive to pH_o_. However, it is possible that changes in pH_o_, in the presence of membrane permeant weak acid such as CO_2_/HCO_3_^−^ might influence vesicle release (Sandstrom, 2011) through changes in pH_i_. Application of the weak acid propionate, known to acidify cells (Sharp and Thomas, 1981) including the NMJ (Lindgren et al., 1997), caused a rise in mEPP frequency which was enhanced and prolonged by EIPA, an inhibitor of NHE. Indeed, EIPA alone provoked an increase in mEPP frequency. NH_4_^+^ removal (Boron and De Weer, 1976) is known to cause NMJ acidification (Chen et al., 1998; Lindgren et al., 1997) of a similar size to the activity-induced pH_i_ shifts (Rossano et al., 2013) and resulted in a transient mEPP frequency increase that was also prolonged by EIPA. Since *Drosophila* muscle is known to have a degree of electrical coupling (Ueda and Kidokoro, 1996), we considered the possibility that the mEPP frequency changes might be due to changes in coupling between neighbouring cells; however, frequency distributions of mEPP amplitudes did not reveal a rise in small mEPP amplitudes during periods of elevated mEPP frequency and in voltage-clamp experiments, while holding at hyperpolarized potentials, there was no significant increase in holding current associated with periods of raised mEPPs which might suggest low-resistance coupling to neighbouring cells of more depolarized potentials. Furthermore, the mEPP and mEPC detection technique is relatively insensitive to the slow and small miniature events that might spread from neighbouring muscle cells. Thus, these results are most easily explained by a pH_i_ sensitive step in vesicle fusion.

It is unlikely that the effects of EIPA seen here were through an action on acid-sensing ion channels since they do not seem to be present at the *Drosophila* NMJ (Sandstrom, 2011), and in the preparations where they do occur, they are blocked by low concentrations of amiloride (*K*_d_ ∼ 10 µM; Waldmann et al., 1997), concentrations at which we saw no effects (data not shown). EIPA also did not appear to block glutamate receptors, since it had no effect on the size of the mEPPs while it increased mEPP frequency. Calcium-independent modulations of mEPP frequency have been reported (Cohen and Van Der Kloot, 1976; Otsu and Murphy, 2003; Rojas et al., 2003) with the suggestion that the acidification-induced rise in mEPP frequency might be caused by the titration of surface charges on the vesicle or inner face of the plasma membrane lowering the energy barrier for fusion. Such pH-dependent vesicle fusion has been reported in other preparations including kidney tubules (Schwartz and Al-Awqati, 1985) and may occur independently of membrane proteins (Minkenberg et al., 2011).

The effect of EIPA was to increase both the amplitude and duration of the mEPP frequency rise during intracellular acidification (propionate or NH_4_^+^ removal) confirming the previous findings that NHE is a regulator of presynaptic pH_i_. Since EIPA alone increases mEPP frequency, it seems likely that NHE is active at steady state and that vesicle release is sensitive to changes of pH_i_ close to the physiological range such that even small pH_i_ shifts might influence vesicle release. Such pH_i_ changes would occur as a result of the counter-transport of H^+^ on the PMCA following a rise in intraterminal calcium. However, they may also result from fluctuations in weak acids or bases concentrations as a result of vesicles releasing their contents into the synaptic cleft.

### Positive-feedback potentiation by exocytosis

Oscillations in evoked transmitter release (in 0 Ca^2+^) and mEPP frequency have been previously reported at the frog NMJ (Meiri and Rahamimoff, 1978; Pawson and Grinnell, 1989); however, the mechanism underlying them remains unexplained.

Following vesicle fusion, the exocytosed contents can transiently change the concentration of acid equivalents within the cleft. If these acid equivalents can cross the pre-synaptic membrane fast enough (e.g., ammonium diffusion coefficient ∼40 nm ms^−1^; Swietach et al., 2005), a pre-synaptic acidification may occur. This acidification would then cause more fusion resulting in positive feedback causing the fusion rate to rise. The process would be limiting in a number of ways. Either the availability of releasable vesicles declines as the release rate rises, and/or the equilibria for NH_3_ shift, such that the driving force for NH_3_ exit declines. Any subsequent reduction in fusion rate (causing less of a cleft acidification) would result in a reversal of the gradients for NH_3_ flux and a pH_i_ increase giving rise to a positive feedback decay in fusion frequency. Since there are three compartments—cleft, vesicle, and cytoplasm—each with different pH, weak acid/base concentration, buffering power, and diffusion dynamics, it is difficult to predict the transmembrane acid fluxes. Nevertheless, once mEPP frequency rises, [NH_3_] equilibria must depart from steady state and the resultant effect must also be near membrane pH changes. Since the release process involves the movement of net acid out of a vesicular compartment in the NMJ, there must, after release, exist a thermodynamic driving force for acid re-entry.

If such a positive feedback were to occur, then it should be abolished under conditions where no net acid efflux occurs. This is consistent with Sandstrom's (2011) result, in *Drosophila* NMJ, where a reduction in evoked quanta occurred at acidic pH_o_, an observation which could not be ascribed to previously described extracellular pH-sensitive targets. While negative feedback effects for both local pH_i_ transients (Behrendorff et al., 2010; Tombaugh, 1998; Xiong et al., 2000) and cleft pH transients (DeVries, 2001; Palmer et al., 2003) have been shown, mostly through an effect on intracellular calcium, we raise the possibility that acid equivalents released from fusing vesicles may also have a positive-feedback role. The known negative feedback roles for protons and this potential positive-feedback role are not mutually exclusive or contradictory. Indeed, they may act together with the acidification to ensure appropriate transmitter release while minimizing calcium influx. Our data do not address whether cleft pH has other effects on exocytosis, including alterations in calcium influx or neurotransmitter receptor sensitivity.

The stimulation-evoked pH_i_ shifts, rise in mEPP frequency on intracellular acidification and positive-feedback effect of released acid raise the possibility that a portion of NMJ transmission might involve pH_i_ changes. The calcium-dependent pH_i_ shift would be predicted to increase fusion probability while any positive feedback of acid may recruit further vesicle release.

The process predicts two phases of vesicle release: an initial PMCA-induced pH_i_ change evoking release followed by a slower feedback of vesicular acid—perhaps similar to the synchronous and asynchronous phases that have been reported (Xue et al., 2011). Such a sequence is consistent with the observations that calcium buffers, which release H^+^ on binding calcium (e.g., EGTA), can be relatively ineffective at inhibiting vesicle release and that a change in the inactivation kinetics of calcium channels can result in little change in vesicle fusion. There are many other potential targets for acid released from vesicles and the interaction between the known negative-feedback effects (Palmer et al., 2003) and this potential positive feedback is unknown. Although a plethora of exocytotic pathways exist, it is not yet clear whether physiologically relevant pH changes target only a sub-set of them. However, such pH-feedback would provide an additional mechanism to explain excitability changes caused by agents that can modulate pH dynamics through effects on H^+^ buffering such as CO_2_ anaesthesia, ketogenic diets and hyperventilation. Those attempting to explain how excitability changes result from hyperventilation have struggled to find a protein with sufficient pH sensitivity (Somjen and Tombaugh, 1998). The pH_i_ sensitivity of vesicle release, with positive feedback, might act as a high gain pH-sensor and help explain such profound excitability changes.
